# Comparison of paraffin-embedded skin biopsies from different anatomical regions as sampling methods for detection of *Leishmania *infection in dogs using histological, immunohistochemical and PCR methods

**DOI:** 10.1186/1746-6148-2-17

**Published:** 2006-06-08

**Authors:** Sílvio Coura Xavier, Hélida Monteiro de Andrade, Semíramis Jamil Hadad Monte, Ingrid Maria Chiarelli, Wanderson Geraldo Lima, Marilene Suzan Marques Michalick, Washington Luiz Tafuri, Wagner Luiz Tafuri

**Affiliations:** 1 Departamento de Clínica e Cirurgia, Escola de Veterinária, Universidade Federal de Minas Gerais, Belo Horizonte, Brasil; 2 Departamento de Parasitologia, Laboratório de Biologia Molecular e Imunogenética, Universidade Federal do Piauí, Teresina, Brasil; 3 Departamento de Patologia Geral, Instituto de Ciências Biológicas, Universidade Federal de Minas Gerais, Belo Horizonte, Brasil; 4 Departamento de Anatomia Patológica, Faculdade de Medicina, Universidade Federal de Minas Gerais, Belo Horizonte, Brasil; 5 Departamento de Parasitologia, Instituto de Ciências Biológicas, Universidade Federal de Minas Gerais, Belo Horizonte-MG, Brasil

## Abstract

**Background:**

We compared skin biopsy samples from different anatomical regions for detecting *Leishmania *in dogs, using histological (HE), immunohistochemical (IHC) and polymerase chain reaction (PCR) techniques.

**Results:**

The sensitivity was 82.8 percent for PCR, 62.1 percent for IHC and 44.8 percent for HE. These methods do not appear to depend on the clinical status of the animal or the anatomical source of the skin sample; there is no "best region" for any method. However, PCR was more effective than IHC and HE for ear and nose skin samples whereas IHC was better than HE for nose samples. There was weak agreement between PCR and HE for all tissue samples; good agreement between PCR and IHC for ear and abdomen samples, and weak agreement for nose; and optimal agreement between IHC and HE for ear and abdomen and good agreement for nose samples.

**Conclusion:**

The PCR on ear skin could be the best procedure for diagnosing canine visceral leishmaniasis. The good agreement between PCR and IHC indicates that IHC can be used as an alternative method. Finally, tissue samples from ears, nose and abdomen, particularly ears and nose, are potentially useful for diagnosing canine visceral leishmaniasis independently of the animal's clinical status.

## Background

Leishmaniasis is endemic in many areas of tropical and subtropical America [at least 24 countries], where it constitutes a significant public health problem. The disease in this region is basically a zoonosis; humans are only incidental hosts in the life cycles of the various pathogenic parasite species [11]. In America, Zoonotic Visceral Leishmaniasis [ZVL] is caused by *Leishmania infantum *(syn. *L. chagasi*) [20], and domestic dogs (*Canis familiaris*) are established as reservoir hosts. Visceral Leishmaniasis is endemic in European, Asiatic and Africa countries, and new areas in which the infection is being disseminated are being identified [9,12,21]. Canine Visceral Leishmaniasis (CVL) exists in about 50 of the 88 countries in which human leishmaniasis is present, and there are three major foci: China, the Mediterranean Basin and Brazil [3].

Control of leishmaniasis in the New World is complicated by the variety of different *Leishmania *species and their diverse clinical manifestations, and by the fact that each parasite species has a unique epidemiological pattern. Since a combined risk exists for both canine and human infections in areas where ZVL is endemic, there is a need for sensitive and specific diagnostic techniques [11]. Serological testing can identify exposure to the parasite but cannot indicate an active infection. Some years ago, the reference standard for diagnosis was the demonstration of parasite by either microscopy or culture of aspirates from spleen, lymph nodes or bone marrow. However, the overall sensitivity of these methods in humans and dogs is variable and relatively poor [24]. Direct parasite detection on histological skin biopsies, which can be obtained by an extremely simple surgical procedure, is a good tool for a definitive diagnosis and for clinical follow-up of dogs undergoing treatment. However, examination of routinely prepared histological sections stained with Hematoxylin and Eosin [HE] is frequently inconclusive, particularly in the skin [6,10,29]. Immunohistochemical detection of *Leishmania *amastigotes in formalin-fixed, paraffin-embedded sections of canine [6,10] and human [15] tissues has previously been described and is routinely used in many laboratories. Recently, an alternative immunohistochemical method was described that could be a useful tool for epidemiological, clinical and histopathological studies [30]. In the last decade, the polymerase chain reaction (PCR) was shown to be sensitive and specific for demonstrating *Leishmania *DNA. In recent years, PCR has considerably improved the diagnosis of canine leishmaniasis, showing 89 to 100% sensitivity in symptomatically or parasitologically confirmed cases [3]. A variety of canine tissues including bone marrow, spleen, lymph nodes, skin and conjunctival biopsy specimens have been used for diagnosis. Blood and bone marrow are the most frequently used tissues for PCR diagnosis in dogs; however, the skin is considered an important tissue reservoir of parasites in both healthy and sick *Leishmania-*infected dogs [1,25,26]. Moreover, positive PCR has been shown to be a better indicator in skin (51%) than in bone marrow (17.8%) or conjunctiva (32%) [25]. PCR using noninvasively obtained samples will be useful for epidemiological studies and for direct diagnosis of canine visceral leishmaniasis [8,27].

The aim of this study was to compare three indirect methods for detecting *Leishmania *in canine skin biopsies obtained from different anatomical regions. Histopathological (HE), immunohistochemical (IHC) and polymerase chain reaction (PCR) methods for detection of *Leishmania *were analyzed and their relative efficacies were considered.

## Results

**Figure 1(a):**
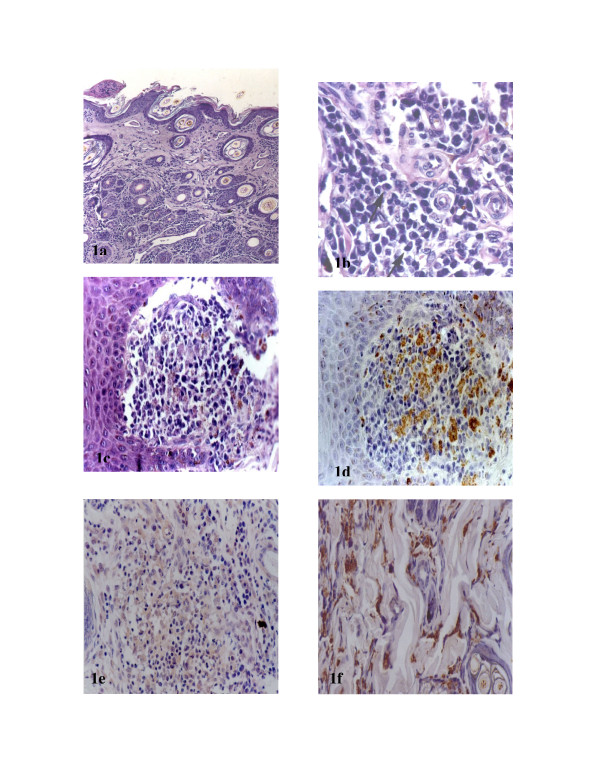
Ear skin section of naturally infected animal. Observe a diffuse inflammatory cellular exudate in up dermis and focal around vessels, pylous and glands of deep dermis. HE 40×. **B **Ear skin section of naturally infected animal. Observe a diffuse inflammatory mononuclear exudate (arrows showing plasmocytes) in a dermis. HE 440×. **C **Nose skin section of naturally infected animal. Observe an intense inflammatory exudate of mononuclear cells in a dermis. HE 110×. **D **Nose skin section of naturally infected animal. Observe innumerous imunolabelled amastigotes forms of *Leishmania*. Streptoavidin-peroxidase method. 110×. **E **Ear skin section of symptomatic naturally infected animal. Intense inflammatory exudate can be observed. Note the absence of amastigotes forms of *Leishmania *HE 440×. **F **Ear skin section of asymptomatic naturally infected animal. Innumerous amastigotes of *Leishmania *can be observed. Note the absence of inflammatory exudate. HE 440×.

**Figure 2:**
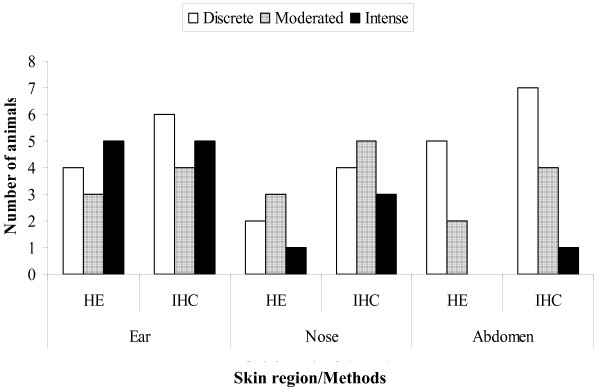
*Leishmania *amastigotes visualized by histopathological (HE) and immunohistochemical (ICH) methods in skin biopsies. Parasitism load classified as discrete, moderated and intense.

Histologically, skin samples showed a chronic inflammatory reaction irrespective of anatomical region, but this reaction varied with the animal's clinical status. In general, the reaction ranged in intensity from discrete to moderate. However, an intense inflammatory process was more frequent in some cases. It was detected only in ear skin biopsies from oligosymptomatic and symptomatic animals. In general, the chronic inflammatory reaction was characterized by a mononuclear infiltrate and was diffuse in the upper dermis and focal around vessels, pyli and glands of the deep dermis [fig. 1(a), 1(b)]. Parasites were more readily identified in ear biopsies than in biopsies from nose and abdominal samples, but the parasite load determined by optical microscopy (HE) showed no statistical differences. However, there was a tendency towards a higher parasite load in ear skin tissue specimens than in the others (data not shown). Numerous imunolabeled amastigote forms of *Leishmania *were readily observed in skin tissues. Moreover, the IHC method improved the HE parasite load data [figs 1(c), 1(d) and figure 2]. In addition, when we compared the parasite load with the definite clinical status of the animal (without parasite load classification), the numbers of positive symptomatic dogs were higher than asymptomatic and oligosymptomatic ones.

Like the HE results, the IHC parasite tissue load data showed no clear relationship to the inflammatory infiltrate. Moreover, we found symptomatic animals with an intense inflammatory infiltrate but without amastigotes, and asymptomatic animals with a discrete inflammatory reaction but many macrophages loaded with amastigotes [Figs 1(e), 1(f)].

The sensitivities of the tests used were calculated using optical microscopy (OM) as gold standard. All 29 animals were OM positive. PCR was positive in 24 dogs, IHC in 18 and HE in 13. The sensitivities were therefore 82.76% for PCR, 62.07% for IHC and 44.83% for HE.

When we compared the PCR, IHC and HE methods according clinical status using ear, nose and abdomen skin samples, there was higher positivity for PCR than IHC and HE, respectively, for all clinical groups. The asymptomatic positivity was higher for PCR method. However, the sample size was not large enough to qualify for statistical significance (Table 1).

In according to Table 2, considering the three methods together for *Leishmania *detection with skin biopsies from different anatomical regions (each line) we found the following results: (1) PCR is better than IHC and HE for ear skin biopsies (p < 0.05); (2) nose skin biopsies give statistically different results for all diagnostic methods, PCR detecting more positive cases followed by IHC and HE; (3) abdominal biopsies show no statistically significant results for any of the three methods.

In addition, the comparison of the skin biopsies from different anatomical regions for each diagnosis method (each column on Table 2) did not show any statistical differences. This means that one anatomical skin region is no more useful than any other for a given test method. Index κ was used to calculate the agreement among the three tests. IHC and PCR agreement well for ear (κ = 0.5) and abdomen (κ = 0.4) skin tissues, but weakly for nose (κ = 0.2). PCR and HE agreement weakly for ear (κ = 0.3), abdomen (κ = 0.2) and nose (κ = 0.05). IHC and HE agreement optimally for ear (κ = 0.8) and abdomen (κ = 0.8) and showed good agreement for nose (κ = 0.5).

## Discussion

Serological techniques for diagnosing *Leishmania *such as the immunofluorescence antibody test (IFAT), direct agglutination test (DAT), enzyme-linked immunosorbent assay (ELISA), dot-ELISA and Western blot are widely used. Although they are clinically very useful, these methods underestimate the infection rate of *Leishmania *in dog populations in endemic areas [3,22]. For canine visceral leishmaniasis, no technique is 100% sensitive and specific and can serve as "gold standard" for diagnosis [3], but direct observation of the parasite in stained smears or tissue sections must be regarded as conclusive. In the last decade, PCR has had the greatest success because of its high sensitivity and specificity [5,19]. PCR can be carried out using genomic or kinetoplast parasite DNA on blood, skin, lymph node, conjunctiva or bone marrow samples from infected animals. However, although blood and bone-marrow are mostly used, skin merits special consideration. Dogs naturally infected with *L. infantum *harbor high numbers of parasites in skin irrespective of the presence of lesions [1,25]. Moreover, skin tissue is a good substrate for PCR diagnosis [18].

Few publications have considered application of PCR to different canine tissues for detecting *Leishmania *infection. Solano-Gallego et al. (2001) [25] showed that 51% of positive animals were identified from skin analysis versus 32% from conjunctiva and 17.8% from bone marrow. Moreover, PCR results from specimens of skin have demonstrated higher sensitivity (87.2%) than spleen (84.6%), liver (80%), lymph nodes (76.9%) or bone marrow (66.7%) [4]. In this work we considered HE, IHC and PCR methods for detecting *Leishmania *in three different skin biopsies (ears, nose and abdomen). All samples utilized were embedded in paraffin and maintained at room temperature. This is important, especially for PCR, because it works even after a long storage time. Based on the MO method as "gold standard" for diagnosis, the sensitivities of the three methods were: PCR 82.8%, IHC 62.1% and HE 44.8%. The PCR sensitivity value accords with previous work: 60% [19], 71.4% [4], 87% [16] and up to 100% [5]. We have not found IHC or HE sensitivity values in the literature. The use of IHC to detect amastigotes in canine tissues has been reported previously and has undoubtedly proved efficient for diagnosis. In this paper we employed a straightforward and inexpensive immunohistochemical approach for detecting *Leishmania *in formalin-fixed, paraffin-embedded canine tissues [30]. We still do not have conclusive results for absolute sensitivity values, but our data show an increase in the number of positive animals [parasite detected] when IHC is compared to HE. Indeed, Bourdoiseau et al. (1997) [6] reported that HE is a good tool for describing lesions, but has low sensitivity for detecting *Leishmania*.

In according to Table 1 PCR was higher positive absolute numbers in asymptomatic and oligosymptomatic dogs and PCR, HE or IHC was independent of the clinical status and tissue origin. However, our sample size was not large enough to qualify for statistical significance. Thus, we did not find statistical difference among the three methods in respect of the clinical status of the animals or the anatomical region from which the skin biopsy was taken. This could indicates that asymptomatic dogs have parasites in the skin just as symptomatic dogs do, underlining the importance of asymptomatic dogs in the epidemiology of visceral leishmaniasis, as discussed by Abranches et al. (1998) [2] in Portugal, Solano-Gallego et al. (2001) [25] in Spain and Lima et al. (2004) [14] in Brazil. Solano-Gallego et al. (2001) [25], for example, showed that PCR was able to detect subclinical canine *Leishmania *infection.

PCR, IHC and HE were simultaneously studied in the three different canine skin samples. PCR was better than IHC and HE for ear and nose skin samples. As PCR method is able to demonstrate *Leishmania *DNA in the investigate samples, it could explained these data.

Optical microscopy revealed parasites (IHC and HE) easier in ear skin specimens than in nose or abdomen samples. In addition, the chronic inflammatory reaction in ear samples was more intense in oligosymptomatic and symptomatic dogs. On the other hand, PCR detected higher numbers of positive animals, followed by IHC and HE; in this case, IHC was better than HE. It is important to say that IHC improved the detection of positive infection by almost fifty percent over HE, as depicted in fig. 1(c), 1(d). PCR, IHC and HE data on the abdomen skin samples showed no statistical differences. These results corroborated the HE and IHC results, because abdomen skin specimens showed lower inflammation and parasite load in all cases.

## Conclusion

Taking these results together, we can suggest that PCR was the best method for diagnosing canine infection by *Leishmania*. However, we can not infer what skin anatomical regions is the best for diagnosing the infection. On the other side, ear skin biopsies is a non-invasive procedure and easier to perform than skin biopsies of other anatomical regions. Moreover, it is less blood-irrigated and the anatomical location is more comfortable for both the animal and the veterinary practitioner. Thus, we think that PCR skin ear biopsy could be used as the best method for diagnosing canine infection by *Leishmania*. However, precautions have to be taken in order to interpret PCR results as a signal of animal infectivity, because PCR positive detection does not necessarily indicate alive parasites in tissues. Until now it has not be demonstrated that all PCR positive dogs are infective.

The good agreement between PCR and IHC indicates that IHC can be used as an alternative method. Finally, skin samples from ears, nose and abdomen, mainly ears and nose, are a potentially useful tissue source for diagnosing canine visceral leishmaniasis independently of the animal's clinical status.

## Methods

### Animals

Twenty-nine mongrel dogs of unknown age naturally infected with *Leishmania *were identified during an epidemiological survey of canine visceral leishmaniasis carried out by the municipality (Zoonosis Department) of Belo Horizonte, MG, (Brazil Southeastern). The tests used were indirect immunofluorescence antibody titres (IFAT), complement fixation reaction (CFR), and enzyme-linked immunosorbent assay (ELISA). Immunofluorescent titres (> 1:40 dilutions), CFR (> 1:40 dilutions) and ELISA (Optical Density > 100; > 1:400 dilutions) were positive (IgG) for all animals. In addition, we tested with a commercial kit containing an immunochromatographic strip that uses recombinant leishmanial antigen k39. This is a dominant amastigote antigen of *Leishmania chagasi *(rK39) and is highly sensitive and specific for *Leishmania donovani *complex infection, as previously described [7,13,28]. Sera from all infected dogs were also positive for this test. All infected dogs were clinically classified according to a previously reported study [14,17], as follows: asymptomatic apparently healthy animals (n = 10), oligosymptomatic animals exhibiting some clinical signs of the disease and/or lesions such as lymphoid adenopathy, moderate weight loss and/or dull brittle hair accompanied by cutaneous lesions (n = 10) and symptomatic animals that exhibited the classical signs of the disease such as cutaneous alterations (alopecia, dry exfoliative dermatitis or ulcers), onychogryphosis, keratoconjunctivitis, cachexia and anemia (n = 9).

### Necropsy, parasitological diagnosis and histopathology

Dogs were sacrificed with a lethal dose of 2,5% (1,0 ml/Kg) Thiopental (intravenous) and and T61™ (0,3 ml/Kg). During necropsy, tissue touch preparations (smears) of liver, spleen and lymph node samples were obtained to confirm *Leishmania *infection. The smears were air-dried and Giemsa stained. The presence of *Leishmania *amastigotes was detected in all animals by light microscopy using oil immersion (1000 × magnification). In addition, livers and spleens specimens were collected from all the dogs for histopathology. Amastigote forms of *Leishmania *could be observed inside macrophages in all the livers and spleens. Samples skin tissue from ear, nose and abdomen were collected and fixed in 10% neutral buffered formalin. All tissue samples were dehydrated, cleared, embedded in paraffin, cut into 4–5 μm thick sections and stained with Hematoxylin and Eosin (HE). We classified all skin samples in terms of chronic dermal inflammation, grading this reaction as discrete (a diffuse inflammatory mononuclear cell infiltrate in the upper dermis), moderate (a diffuse and localized cellular infiltrate around glands and vessels in the deep dermis) or intense (a diffuse cellular infiltrate in all dermis layers).

### Immunohistochemical (IHC) method for labeling amastigotes of Leishmania

Deparaffined slides were hydrated and incubated in 4% hydrogen peroxide (30 v/v) in 0.01 M PBS, pH 7.2, followed by incubation with normal goat serum (diluted 1:100). A heterologous immune serum from dogs naturally infected with *L. chagasi *(diluted 1:100 in 0.01 M PBS) was used as primary antibody. Slides were incubated for 18–22 h at 4°C in a humid chamber. After washing in PBS, the slides were incubated with biotinylated goat anti-mouse and anti-rabbit (Link-DAKO, LSAB2 kit, California, USA), washed again in PBS and incubated with streptavidin-peroxidase complex (Link-DAKO, LSAB2 kit, California, USA) for 20 min at room temperature. The reaction was developed with 0.024% diaminobenzidine (DAB; Sigma, St Louis, USA) and 0.16% hydrogen peroxide (40 v/v). Finally, the slides were dehydrated, cleared, counter-stained with Harris's hematoxylin and mounted with coverslips [30].

The skin tissue parasite loads were analyzed by the presence of immunolabeled amastigote forms of *Leishmania *associated with the chronic inflammatory reaction in the dermis. A semi-quantitative study was carried out by optical microscopy (110×). Parasite load was measured as: (+) discrete, (+) moderate and (+++) intense.

### Polymerase chain reaction (PCR)

Initially, the samples were washed in ethanol then xylol to dissolve the paraffin, then DNA was extracted from a skin specimen measuring approximately 1.0 mm^3 ^by soaking in TE buffer (10 mM Tris-HCl, 1 mM EDTA, pH 7.5) and incubating with proteinase K (Life Technologies, USA) at a final concentration of 100 μg/ml for 3 h at 56°C. The proteinase was inactivated by heating at 100°C for 15 min. The lyses obtained were processed using a Wizard TM Genomic Purification Kit (Promega, Madison, WI) according to the manufacturer's instructions. Two primers were used to amplify a specimen of the conserved region of *Leishmania *kDNA minicircles (5'-GGG [G/T]AGGGGCGTTCT [G/C]CGAA-3' and 5'- [G/C] [G/C] [G/C) (A/T]CTAT [A/T]TTACACCAACCCC-3'). A 120-base pair (bp) PCR product was generated (Degrave et al., 1994). Reactions were carried out in volumes of 10 μl containing 1.0 μg of DNA, 0.2 mM dNTPs, 10 mM Tris-HCl (pH 8.0), 50 mM KCl, 1.5 mM MgCl_2_, 5 pmol of each primer and 1 U *Taq *polymerase. The conditions for PCR amplification were as follows: initial denaturation at 94°C for 5 min, 30 cycles consisting of denaturation at 94°C for 1 min, annealing at 65°C for 1 min and extension at 72°C for 1 min, and final extension at 72°C for 5 min. The reaction mixtures were cycled in a Perkin-Elmer GenAmp 9600 thermocycler. In all assays, a positive control containing *L. chagasi *(MHOM/BR/1972/BH46 strain) genomic DNA and a negative control without DNA were included. Following amplification, the samples were submitted to electrophoresis on 6% polyacrylamide gel and silver-stained. The DNA of bacteriophage φX 174 cleaved by HAE III was used as a molecular marker (Pharmacia, Upsala, Sweden).

### Statistical analysis

Descriptive analysis were applied for histological and parasite load results. Inference analysis was carried out by chi-square test (software SPSS 1.1 Windows version) for the three methods (HE, IHC and PCR) and the different skin anatomical regions studied. A *p *value of less than 0.05 was considered significant. As a measure of agreement, index Kappa (κ) was also evaluated between PCR × IHC, PCR × HE and HE × IHC. We accepted κ <0.4 as weak agreement, 0.7> κ >0.4 as good agreement and κ > 0.7 as optimal agreement. The sensitivity of the test was determined using optical microscopy (OM) as the gold standard [23].

## Authors' contributions

SCX, IMC, WGL and WLT: Carried out the skin necropsies, histopathology and immunocytochemistry

HMA and SJHM: Carried out the PCR and all the statistical analysis

MSMM: Carried out the serological tests [CRF, ELISA, IFAT, TRALd] for *Leishmania *infection

HMA, WLT and WLT have made substantial contributions to conception and design, analysis and interpretation of data

## Figures and Tables

**Table 1:** Comparison of *Leishmania *detection by polymerase chain reaction (PCR), immunohistochemical (IHC) and histopathological (HE) methods in biopsies from distinct skin anatomical locations according to clinical status: asymptomatic (n = 10), oligosymptomatic (n = 10) and symptomatic (n = 9)

**Method**	**Clinical Group**	**SKIN LOCATION**			**% POS **(positive at last one location)
			
		**Ear**	**Nose**	**Abdomen**	
**PCR**	Asymptomatic	7	6	2	**90%**
	Oligosymptomatic	7	8	5	**80%**
	Symptomatic	7	5	7	**78%**

**IHC**	Asymptomatic	3	4	4	**70%**
	Oligosymptomatic	4	2	2	**40%**
	Symptomatic	7	5	5	**78%**

**HE**	Asymptomatic	3	2	2	**30%**
	Oligosymptomatic	4	1	2	**40%**
	Symptomatic	5	7	1	**67%**

**Table 2:** Comparison among polymerase chain reaction (PCR), immunohistochemical (IHC) and histopathological (HE) methods for *Leishmania *detection versus canine skin biopsies from different anatomical locations in 29 naturally infected dogs

**Skin region**	**PCR +**	**(%)**	**IHC +**	**(%)**	**HE +**	**(%)**
**Ear**	21^a^	(72.4%)	14^b^	(48.3%)	12^b^	(41.4%)
**Nose**	19^a^	(65.5%)	11^b^	(37.9%)	5^c^	(17.2%)
**Abdomen**	14^a^	(48.3%)	11^a^	(37.9%)	5^a^	(17.2%)

Each data point is associated a one letter (a, b or c). Data with the same letters means no statistical difference. Otherwise, different letters means statistical difference. Each line: Letters a, b, c, represent statistical analysis considering the three methods together. Different letter were used to significant differences, chi-square test (p < 0.05).
